# A new species of the genus *Kamimuria* (Plecoptera, Perlidae) from Jiangsu, China with re-description of *K.microda* Du, 2002

**DOI:** 10.3897/BDJ.12.e137424

**Published:** 2024-11-18

**Authors:** Liang-Liang Zeng, Qing-Bo Huo, Yu-Zhou Du

**Affiliations:** 1 College of Plant Protection & Institute of Applied Entomology, Yangzhou University, Yangzhou 225009, China College of Plant Protection & Institute of Applied Entomology, Yangzhou University Yangzhou 225009 China; 2 Joint International Research Laboratory of Agriculture and Agri-Product Safety, the Ministry of Education, Yangzhou University, Yangzhou 225009, China Joint International Research Laboratory of Agriculture and Agri-Product Safety, the Ministry of Education, Yangzhou University Yangzhou 225009 China

**Keywords:** Plecoptera, Perlidae, new species, Jiangsu, Guizhou

## Abstract

**Background:**

Jiangsu Province is located on the southeast coast of China and lacks high-altitude mountains, with only a few hilly areas mainly distributed in its southern region. In recent years, We investigated the diversity of Plecoptera species in the major mountainous and forested areas of Jiangsu, recording a total of 20 species across 12 genera and five families.

**New information:**

In this paper, we examined the materials of *Kamimuria* from Jiangsu and described a new species, *K.liyangensis* **sp. nov.** In addition, detailed English descriptions and colour pictures of *K.microda* are provided for the first time.

## Introduction

*Kamimuria* Klapálek, 1907 is the most species-rich genus in the subfamily Perlinae with approximately 93 known species worldwide, distributed in the Oriental Realm and Palaearctic Realm, primarily in China (DeWalt et al. 2023). Over the past 30 years, the recorded number of *Kamimuria* species in China has dramatically increased to 60 (Du 1999, Yang and Li 2018, DeWalt et al. 2023, Huo 2023). Jiangsu Province is located on the southeast coast of China and lacks high-altitude mountains, with only a few hilly areas mainly distributed in its southern region. The earliest record of Plecoptera in Jiangsu was made by Wu (1926), who discovered two species of the *Nemouridae* Newman 1853 in Nanjing. Later, Du and Chen (2016), Chen and Du (2015) and Chen and Du 2016 described three new species from Jiangsu. Huo (2019) investigated the diversity of Plecoptera species in the major mountainous and forested areas of Jiangsu, recording a total of 20 species across 12 genera and five families. Recently we examined the materials of *Kamimuria* from Jiangsu and described a new species, *K.liyangensis* sp. nov.

Another species of *Kamimuria* from Guizhou, China, *K.microda* Du, 2002 was published, its morphological characteristics were briefly described only in Chinese and the original illustrations were somewhat unclear (Du and Ran 2002). The presence of this species has not been reported for 22 years and these factors have posed challenges for subsequent identification efforts. After a thorough examination of the type materials of *K.microda*, we re-described the species and provided clear colour photographs for the first time, aiming to facilitate future identification efforts.

## Materials and methods

Specimens were collected by light trap. All materials were preserved in 75% ethanol and the penis were everted using the cold maceration technique of Zwick (1983). Photographs were taken with the KEYENCE VHX-5000 system and subsequently optimised in Adobe Photoshop CS6. All specimens were deposited in the Insect Collection of Yangzhou university (ICYZU), Jiangsu Province, China. Terminology followed Sivec and Stark (2008) and Zwick 2023

## Taxon treatments

### Kamimuria liyangensis sp. nov.

1E664398-5BF5-5217-A11C-EC82D222B2C7

1F9CAA08-2593-43C7-AC92-977D0FCBB133

#### Materials

- **Type status:** Holotype. **Occurrence:** recordedBy: Qing-Bo Huo, Peng Gao; individualCount: 1; sex: male; lifeStage: adult; **Taxon:** kingdom: Animalia; phylum: Arthropoda; class: Insecta; order: Plecoptera; family: Perlidae; genus: Kamimuria; **Location:** country: China; stateProvince: Jiangsu Province; county: Liyang; locality: “Nanshan Bamboo Sea”; **Identification:** identifiedBy: Qing-Bo Huo; **Event:** year: 2018; month: 5; day: 28; **Record Level:** institutionID: ICYZU
- **Type status:** Paratype. **Occurrence:** individualCount: 3; sex: male; lifeStage: adult; **Taxon:** kingdom: Animalia; phylum: Arthropoda; class: Insecta; order: Plecoptera; family: Perlidae; genus: Kamimuria; **Location:** country: China; stateProvince: Jiangsu Province; county: Liyang; locality: “Nanshan Bamboo Sea”; **Identification:** identifiedBy: Qing-Bo Huo; **Event:** year: 2018; month: 5; day: 28; **Record Level:** institutionID: ICYZU

#### Description

**Figure 1.**
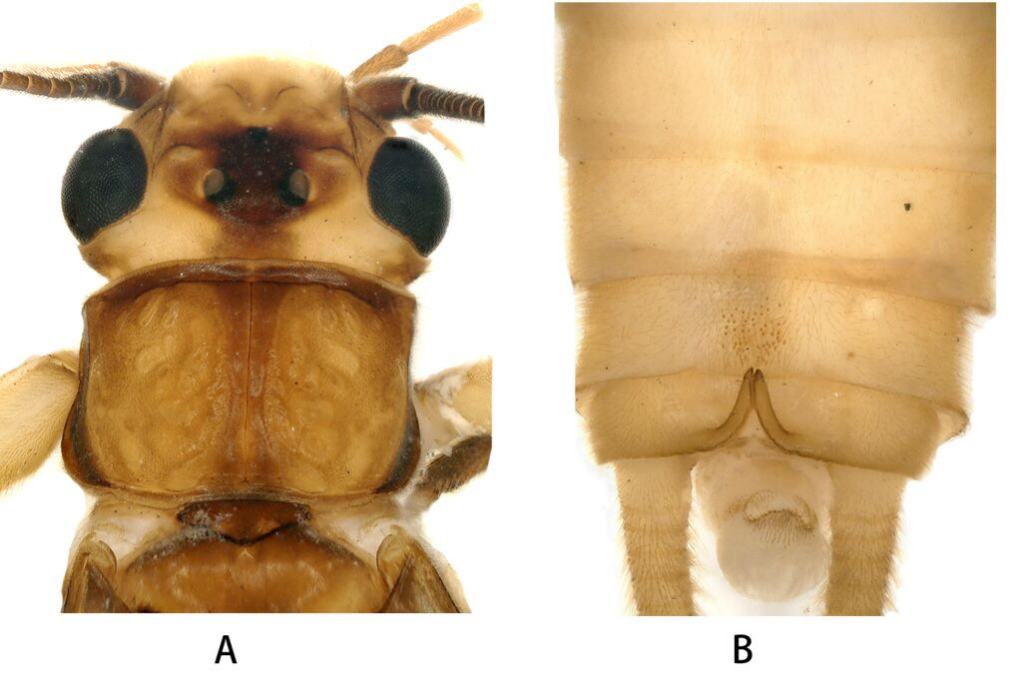
*Kamimurialiyangensis* sp. nov. (holotype, male). **A** head and pronotum, dorsal view; **B** terminalia, dorsal view.

**Male**. Colouration generally brown to dark brown (Due to being preserved in alcohol, the colours in the specimens appear faded in the photograph). Head pale yellow with black marking covering ocellar area (Fig. 1A). Antennae and palpi dark brown. Pronotum dark brown with darker rugosities, anterior margin and stripes along median suture darker. Legs yellow brown with dark knees. Wings membrane grey, veins brown.

Tergum 1–8 unmodified (Fig. 1B). Tergum 9 centre with sensilla basiconica. Hemitergal lobe slender, hook-like, apex slightly re-curved and nearly reaching the posterior margin of tergum 9.

**Figure 2.**
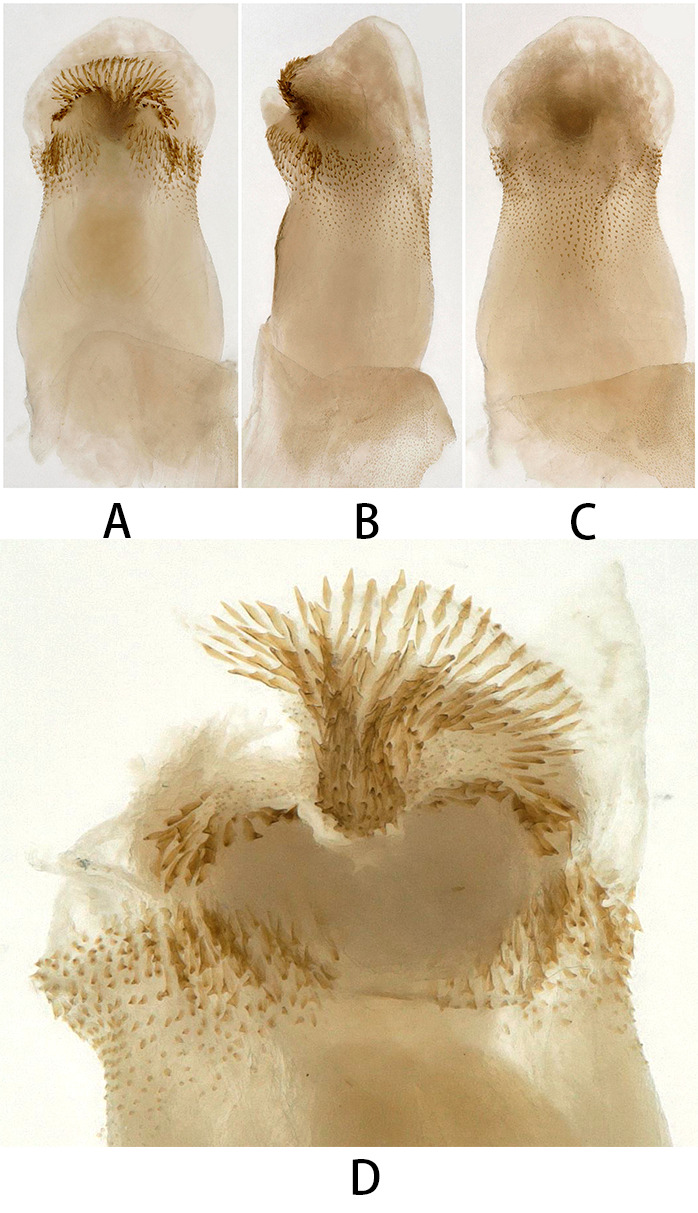
*Kamimurialiyangensis* sp. nov. (holotype, male). **A** penis, ventral view; **B** penis, lateral view; **C** penis, dorsal view; **D** apex of everted endophallus, ventral view.

Penis membranous (Fig. 2), with a ring-shaped group of large spines at the tip, interrupted by a spongy membranous projection in the middle. The base of the penis on the dorsal side is densely covered with small dot-like spines.

**Female, egg and nymph**: Unknown.

#### Etymology

The specific name refers to the type locality, Liyang City.

#### Distribution

This species is known only from the type locality, Liyang City of Jiangsu Province.

#### Taxon discussion

The penis of the new species is similar to that of *K.hainana*, Li, Wang & Yu, 2012 (see figs. 2–10 in Li et al. (2012)). Both species possess a spiny-free membranous region at the apex of the dorsal side of the endophallus, while the base is densely covered with small spines. Additionally, the ventral side of the endophallus features a spiny-free membranous area. However, in *K.hainana*, the basal spinule patch is divided by a funnel-shaped area on the dorsal surface, terminating subapically. The spines on the two lobes form a heart-shaped ring when viewed from the ventral side. In *K.liyangensis*, the dorsal side of the penis base is densely covered with small, dot-like spines. In the ventral view, there is a ring-shaped group of large spines at the tip, interrupted in the centre by a spongy, membranous projection. Currently, this new species is only known to inhabit Liyang City, Jiangsu Province, China. This discovery marks the first record of the *Kamimuria* in Jiangsu Province, enriching the study of insect diversity in the region.

### Kamimuria microda

Du, 2002

B4F8C83C-F7AA-5171-B040-5ED57F59825C

Kamimuria microda Du, 2002 in Li & Jin (2002: 112, 116). China: Guizhou: Maolan, Sanchahe.
Kamimuria microda Du, 2002 in Stark & Sivec (2013: 117).
Kamimuria microda Du, 2002 in Yang & Li (2018: 31).

#### Materials

- **Type status:** Holotype. **Occurrence:** recordedBy: Yu-Zhou Du; individualCount: 1; sex: male; lifeStage: adult; **Taxon:** kingdom: Animalia; phylum: Arthropoda; class: Insecta; order: Plecoptera; family: Perlidae; genus: Kamimuria; **Location:** country: China; stateProvince: Guizhou Province; county: Libo; locality: Maolan National Nature Reserve, Sancha River; **Identification:** identifiedBy: Yu-Zhou Du; **Event:** year: 1994; month: 7; day: 8; **Record Level:** institutionID: ICYZU
- **Type status:** Paratype. **Occurrence:** recordedBy: Yu-Zhou Du; individualCount: 4; sex: male; lifeStage: adult; **Taxon:** kingdom: Animalia; phylum: Arthropoda; class: Insecta; order: Plecoptera; family: Perlidae; genus: Kamimuria; **Location:** country: China; stateProvince: Guizhou Province; county: Libo; locality: Maolan National Nature Reserve, Sancha River; **Identification:** identifiedBy: Yu-Zhou Du; **Event:** year: 1994; month: 7; day: 8–12; **Record Level:** institutionID: ICYZU

#### Description

**Figure 3.**
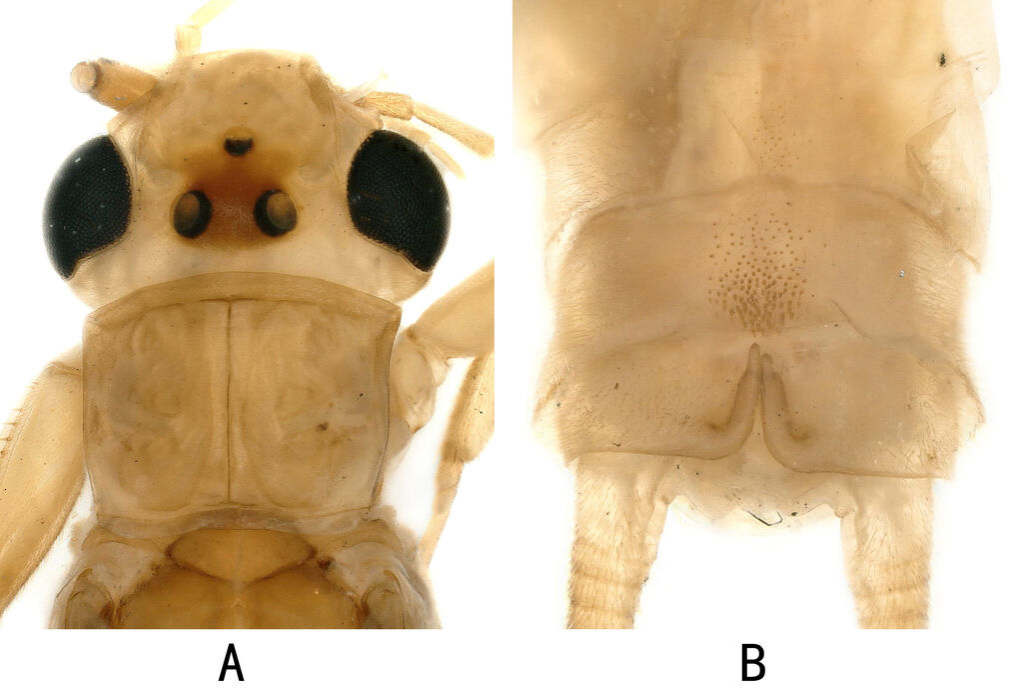
*Kamimuriamicroda* Du, 2002 (paratype, male). **A** head and pronotum, dorsal view; **B** terminalia, dorsal view.

**Male habits**: General colour yellow brown in aged specimens (Fig. 3A). The head is pale yellow with three ocelli, ocellar patch dark brown. The pronotum is nearly horizontally rectangular, with the front angles bluntly pointed and the rear angles rounded, with a rough surface. Abdominal segments and cerci pale brown.

Tergum 1–8 unmodified. Tergum 8 sometimes features a few sensilla basiconica in its central region, Tergum 9 has sensilla basiconica extending up to 1/3 of the anterior margin (Fig. 3B). The inner surface of the hemitergal lobe is flat, extending to 1/3 of the way to the anterior margin of Tergum 10.

**Figure 4.**
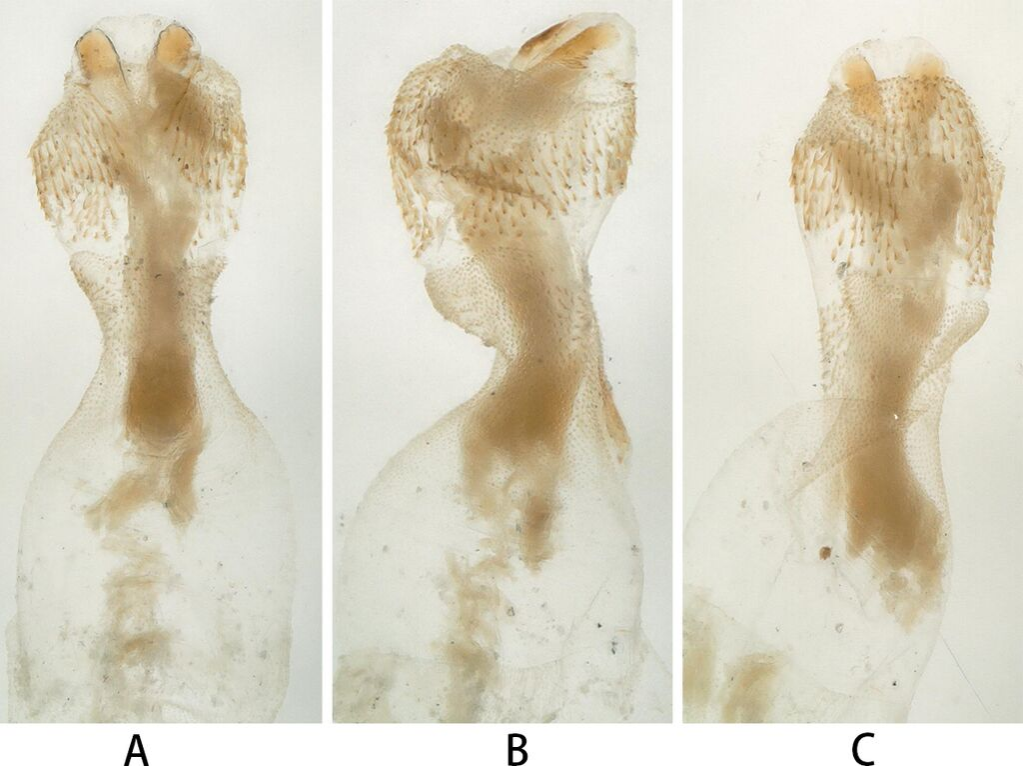
*Kamimuriamicroda* Du, 2002 (paratype, male). **A** penis, dorsal view; **B** penis, lateral view; **C** penis, ventral view.

The base of the penis is bulbous (Fig. 4), tapering anteriorly into a neck-like structure. There are sclerotised patches on the ventral surface of the penis base near its anterior-middle portion and the narrowed distal end of it has numerous very small spines. The endophallus is short sac-like, with small spines densely covering its dorsal and lateral surfaces. There is a pair of sclerites on the ventral surface of the endophallus, with small tooth-like structures on the outer side of the sclerites.

**Female, egg and nymph**: Unknown.

#### Taxon discussion

Due to being preserved in alcohol for nearly 22 years, many features of the type materials have become less distinct. Additionally, original illustrations of the penis of this species are somewhat blurred, posing challenges to identification work. To address this challenge, we photographed the adult male penis from the paratypes, which was in a better preservation condition. These clear images will provide crucial references for further identification work.

## Supplementary Material

XML Treatment for Kamimuria liyangensis

XML Treatment for Kamimuria microda
